# Reprodution of the Crystalline Lens

**Published:** 1867-12

**Authors:** 


*Reproduction of the Crystalline Lens.*—M. Milliot has shown that the crystalline lens is often reproduced after removal from various animals. Similar experiments were tried over forty years ago with the same results.

